# Corneal functional optical zone under monocular and binocular assessment

**DOI:** 10.1186/s40662-018-0097-y

**Published:** 2018-02-07

**Authors:** Samuel Arba Mosquera, Diego de Ortueta, Shwetabh Verma

**Affiliations:** 1 SCHWIND eye-tech-solutions, D-63801 Kleinostheim, Germany; 2 Recognized Research Group in Optical Diagnostic Techniques, University of Valladolid, Valladolid, Spain; 3 Department of Ophthalmology and Sciences of Vision, University of Oviedo, Oviedo, Spain; 4 Augenzentrum Recklinghausen, Recklinghausen, Germany; 5 Experimental Radiation Oncology, University Medical Center Mannheim, Heidelberg University, Mannheim, Germany; 6 Interdisciplinary Center for Scientific Computing (IWR), Heidelberg University, Heidelberg, Germany; 7 Central Institute for Computer Engineering (ZITI), Heidelberg University, Heidelberg, Germany

**Keywords:** Functional optical zone, Bilateral symmetry, Astigmatism, Cardinal and oblique astigmatism, Binocular, Monocular, Intercorneal differences, Correlation, Enantiomorphism

## Abstract

**Background:**

In this retrospective randomized case series, we compared bilateral symmetry between OD and OS eyes, intercorneal differences and Functional Optical Zone (FOZ) of the corneal aberrations.

**Methods:**

Sixty-seven normal subjects (with no ocular pathology) who never had any ocular surgery were bilaterally evaluated at Augenzentrum Recklinghausen (Germany). In all cases, standard examinations and corneal wavefront topography (OPTIKON Scout) were performed. The OD/OS bilateral symmetry was evaluated for corneal wavefront aberrations, and FOZ-values were evaluated from the Root-Mean-Square (RMS) of High-Order Wavefront-Aberration (HOWAb). Moreover, correlations of FOZ, spherical equivalent (SE), astigmatism power, and cardinal and oblique astigmatism for binocular vs. monocular, and binocular vs. intercorneal differences were analyzed.

**Results:**

Mean FOZ was 6.56 ± 1.13 mm monocularly, 6.97 ± 1.34 mm binocularly, and 7.64 ± 1.30 mm intercorneal difference, with all strongly positively correlated, showing that the diameter of glare-free vision is larger in binocular than monocular conditions. Mean SE was 0.78 ± 1.30 D, and the mean astigmatism power (magnitude) was 0.46 ± 0.52 D binocularly. The corresponding monocular values for these metrics were 0.78 ± 1.30 D and 0.53 ± 0.53 D respectively. SE, astigmatism magnitude, cardinal astigmatism component, and FOZ showed a strong correlation and even symmetry; and oblique astigmatism component showed odd symmetry indicating Enantiomorphism between the left and right eye.

**Conclusions:**

These results confirm OD-vs.-OS bilateral symmetry (which influences binocular summation) of HOWAb, FOZ, defocus, astigmatism power, and cardinal and oblique astigmatism. Binocular Functional Optical Zone calculated from corneal wavefront aberrations can be used to optimize refractive surgery design.

## Background

Human vision is a complicated binocular process. Among others, it involves stereopsis, which is the parallax provided by the two eyes’ different positions on the head giving precise depth perception [1], and binocular fusion typified by the visualization of a single image despite each eye having its image of any object [1]. Another vital feature is a binocular summation, which is an enhanced ability to detect faint objects [2] compared to monocular vision.

Howland and Howland, employing the cross-cylinder aberroscope method they invented [3], found that the optical aberrations of the eye differ significantly from subject to subject and are seldom symmetrical. Liang and Williams, using a Shack-Hartmann wavefront sensor [4], found that although the pattern of aberrations varies from subject to subject, the aberrations (regular and irregular) of the left and the right eye of the same subject are correlated, indicating that they are not just random defects. Porter et al. [5] confirmed this observation in a large population.

The Indiana Aberration Study by Thibos et al. [6] characterized the aberration structure and the effects of these aberrations on vision, for a reasonably large population of healthy eyes in young adults, and verified the hypothesis of bilateral symmetry.

Marcos and Burns [7] found that not only aberrations but cone directionality also varies across subjects, but these functions show a left-right eye symmetry. Wang et al. [8] found that even though the wavefront aberrations in the anterior corneal surface vary greatly across subjects, a moderate-to-high degree of mirror symmetry exists between the right and left eyes.

A few studies have addressed the issue of symmetry of aberrations between the right and left eyes of the patients, after corneal laser refractive surgery [9, 10]. Independently developed ray-tracing programs [11, 12] have been used to determine the functional optical zone (FOZ) after the refractive surgery. A direct approach to measure FOZ after refractive surgery has been proposed by manually determining the transition region between the treated and untreated areas from corneal topography maps [13].

The aim of this work was to evaluate the bilateral symmetry regarding corneal wavefront aberrations in 212 non-pathological (normal) eyes (right and left eyes of 106 subjects) that have not undergone any ocular surgery. Wavefront aberrations are often used to describe the optical quality of the eye. We utilized the root mean square (RMS) of the higher-order wavefront aberrations, to define the FOZ in the subjects, allowing a systematic analysis consistent with the formal definitions used to describe aberrations. We analyzed the bilateral symmetry based on corneal wavefront aberration, specifically by correlating the FOZ, defocus, astigmatism power, and cardinal and oblique astigmatism for binocular vs. monocular, and binocular vs. intercorneal differences between the right and left eyes of the same subjects. Several studies justify the equivalence of high order corneal aberrations and high order ocular aberrations [14]. Since the cornea provides the main dioptric power of the eye, we restricted our analysis in this study to anterior corneal wavefront aberrations only.

## Methods

Informed consent was obtained from each patient, for the use of his or her de-identified clinical data for publication. The investigation in this form is not subject to Medical Research Involving Human Subjects Act (WMO). The complete records with the clinical data of 212 eyes of 106 subjects (63 (59%) male and 43 (41%) female subjects) were obtained from the *Augenzentrum Recklinghausen* (Germany). The mean age of the study populations was 32 ± 8 years (range from 19 to 54). We employed SciLab™ (SciLab Enterprises, France, Version 1.0.2) for calculations and running the simulations, Microsoft™ Excel (Microsoft Corporation, US, Version 2010) for plotting graphs, and programming language Delphi (Embarcadero Technologies) for implementing the modules in the Custom Ablation Manager (CAM, SCHWIND eye-tech-solutions GmbH, Version V4.5.31) to analyze the corneal wavefront aberration data. Inclusion criteria for the review were best spectacle corrected distance visual acuity (CDVA) ≥ 20/25 (logMAR ≤0.1) in both eyes, no signs of amblyopia, no previous eye surgery, and a minimum 6.5-mm topographic corneal coverage.

Optical errors centered on the line-of-sight [15] represent the corneal wavefront aberration. These are described by means of Zernike polynomials [16] and their corresponding coefficients, analyzed for a standardized diameter of 6 mm, using OSA standard notations [17] (from which ANSI [18] and ISO [19] standards have been derived). For all eyes, we measured the corneal topography [20] under natural eye conditions without any cycloplegic agents. Corneal wavefront aberrations were calculated from the corneal topography using Fermat’s principle of least time, which is the basis for Snell’s law and refraction [14, 21]. The statistical properties of Zernike expansion have been determined in different studies [22, 23] suggesting that the variance in wavefront aberrations can be identified with reasonable accuracy with the Zernike polynomials up to the seventh Zernike order. Therefore, the corneal wavefront aberrations were fit to the Zernike polynomials up to the 7th Zernike order (36 terms). These calculations were internally performed by the diagnostic device (Keratron-Scout, OPTIKON2000, Rome, Italy, Version 4.6.6 [24]); and corneal topographic data and Zernike coefficients corresponding to and the corneal wavefront aberrations were obtained for each patient. Furthermore, manifest refraction, uncorrected and corrected distance visual acuity (UDVA and CDVA respectively) was measured for each eye.

### Analysis of the functional optical zone

Equivalent defocus means, “the amount of defocusing required to produce the same wavefront variance as found in one or more high-order aberrations” [6]. A simple formula computes equivalent defocus in diopters from the wavefront variance in the Zernike modes in question:

1 $$ {M}_e=\frac{16\sqrt{3} RMS}{PD^2} $$

where M_e_ is the equivalent defocus in diopters, RMS is the root mean square of the higher order wavefront aberrations defining the wavefront variance in the Zernike modes in question, and PD is the diameter considered for the wavefront aberration analysis.

On normal (non-pathological) eyes that have not undergone any ocular surgery, equivalent defocus as proposed by Thibos et al. [6] seems to be relatively insensitive to different analysis diameters. Seiler et al. [25] also described an increase in spherical aberration with pupil dilation in corneas that have undergone photorefractive keratectomy but not in healthy untreated corneas.

Considering this feature of being relatively insensitive to the analysis diameter, we also used root mean square of the higher order aberration (RMSho) to define the FOZ. The Zernike modes higher than the second order were considered as higher orders. By analyzing corneal wavefront aberrations for diameters starting from 4-mm, we increased the analysis diameter in 10 μm steps until reaching the maximum limit of 10 mm. At each step, we iteratively fit the corneal wavefront aberrations to the Zernike polynomials up to the 7th order, until the corneal RMSho was above 0.375 D for the first time. This diameter minus 10 μm (accounting for the last step) was used for determining the FOZ for that case:

2 $$ RMSho(FOZ)=0.375D $$

### Correlations for bilateral symmetry OS vs. OD

We plotted left-vs.-right-eye Scatter graphs for SE, astigmatism magnitude, cardinal and oblique astigmatism components and FOZ, to analyze the predicted correlations between the two eyes. It is expected that SE, astigmatism magnitude, cardinal astigmatism component, and FOZ show even symmetry; and oblique astigmatism component shows odd symmetry. In other words, the left and right eye are symmetric regarding SE, astigmatism magnitude, cardinal astigmatism component and FOZ, with the increasing value of these metrics in one eye also showing an increase in the corresponding eye. Yet, in terms for oblique astigmatism, this would suggest anti-symmetry with an increase in one eye leading to a decrease in the other eye of the subject, substantiating the bilateral symmetry of human vision (or so-called Enantiomorphism).

### Monocular estimator

We defined the monocular estimator as the average of the Zernike components (describing the corneal wavefront aberrations) of OD and the mirrored Zernike components of OS [26] (represented by C_sym(OS)_ below). Therefore, for OS, negative even modes and positive odd modes were sign reversed and averaged with the corresponding Zernike modes of OD:

3 $$ {C}_{MonocularEstimator}\left[n,m\right]=\frac{C_{OD}\left[n,m\right]+{C}_{Sym(OS)}\left[n,m\right]}{2} $$

This criterion estimates the monocular performance of the subjects based on the analyzed metric.

### Correlations for monocular estimator vs. OD and OS

We plotted Monocular estimator vs. left- and right-eye scatter graphs for SE, astigmatism magnitude, cardinal and oblique astigmatism components and FOZ, to analyze the predicted correlations between the two eyes. It is expected that Monocular estimator vs. OD shows even symmetry, and Monocular estimator vs. OS shows odd symmetry.

### Intercorneal difference

We defined the intercorneal difference as the difference of the Zernike components (describing the corneal wavefront aberrations) of OD and the mirrored Zernike components of OS [26] (C_sym(OS)_). Therefore, for OS, negative even modes and positive odd modes were sign reversed and subtracted from the corresponding Zernike modes of OD:

4 $$ {C}_{IntercornealDifference}\left[n,m\right]={C}_{OD}\left[n,m\right]-{C}_{Sym(OS)}\left[n,m\right] $$

This criterion calculates the difference between the right and left eye based on the analyzed metric. The analysis of intercorneal differences based on the FOZ was accounted from the RMS of the differential corneal wavefront aberration (RMS(ΔHOA)).

### Binocular estimator

Jiménez et al. [26] showed the importance of aniseikonia in binocular vision, further suggesting that an increase in the differential ocular aberrations results in reduced bilateral symmetry in the eyes. We defined the binocular estimator based on this implied relation, as the average of the Zernike components (describing the corneal wavefront aberrations) of OD and OS [26–28].

5 $$ {C}_{BinocularEstimator}\left[n,m\right]=\frac{C_{OD}\left[n,m\right]+{C}_{OS}\left[n,m\right]}{2} $$

This criterion estimates the binocular performance of the subjects based on the analyzed metric. These relations (Eqs. 4 and 5) imply that horizontally symmetric higher order aberrations cancel each other to render zero Intercorneal differences and perfect bilateral symmetry.

### Correlations for binocular estimator vs. monocular estimator

We plotted Binocular estimator vs. Monocular estimator scatter graphs for SE, astigmatism magnitude, cardinal and oblique astigmatism components and FOZ to analyze the predicted correlations between the two eyes. This would present a comparison of the estimated binocular vision to the estimated monocular vision, representing the extent of binocular summation.

### Correlations for binocular estimator vs. Intercorneal difference

We plotted Binocular estimator vs. Intercorneal difference scatter graphs for SE, astigmatism magnitude, cardinal and oblique astigmatism components and FOZ to analyze the predicted correlations between the two eyes. This would present how the estimated binocular vision changes with changing differences between the two eyes, representing binocular inhibition through intercorneal differences.

### Description of the search algorithm

Our search algorithm was based on an “increasing diameter” analysis; this ensured that the smallest FOZ satisfying the threshold condition is found. The Zernike fit seems to be less robust for very small analysis diameters, mostly due to the decreasing sampling density within the unit circle. The lower limit of 4 mm FOZ was selected to avoid this error. The higher limit of FOZ corresponding to 0.375 D has been shown to be compatible to CDVA + 0.05 logMAR [29], representing the diameter of glare-free vision. Furthermore, to avoid the flooring and ceiling effects, the eye pairs having calculated FOZ of 4 mm or 10 mm were excluded from our analysis. We did not extend the threshold limits for potentially increasing the sample size, to avoid decreasing the sampling density and to respect the reference limit of CDVA. Therefore, for increasing precision, the datasets where the method reached the equivalent refraction threshold at its very first or very last step were excluded from further analysis.

### Statistical analysis

The various scatter plots were analyzed. These plots shall reveal, for our sample population, the parameters showing symmetry and the type of symmetry associated with them. The slope and intercept of the linear regression (least-square fitting) were calculated for each parameter. We assessed the statistical significance of the correlations using Student’s T-test. The Coefficient of Determination (r^2^) was also employed, and the significance of the correlations was evaluated assuming a metric that is distributed approximately as t with (N-2) degrees of freedom, where N is the size of the sample. The level of statistical significance was taken at *p* < 0.05.

## Results

After excluding the eye pairs with the calculated FOZ of 4 mm or 10 mm, 134 eyes of 67 subjects (40 (60%) male and 27 (40%) female subjects) were further analyzed. In this final sub-cohort of 134 eyes, the mean age was 31 ± 9 years (range from 19 to 54), the mean spherical equivalent (SE) was 0.78 ± 1.30 D (− 4.15 D to + 3.46 D), and the mean cylinder was − 0.57 ± 0.54 D (− 3.41 D to − 0.03 D).

### Corneal wavefront aberration

Average root mean square of the high order wavefront aberration (RMSho) was 0.545 ± 0.136 μm at 6 mm (from 0.327 μm to 0.848 μm). This distribution of corneal aberration in Zernike terms can be regarded as normal [30]. Spherical aberration was 0.376 ± 0.158 μm (from 0.025 μm to 0.744 μm), coma aberration was 0.272 ± 0.140 μm (from 0.030 μm to 0.610 μm), and trefoil aberration was 0.144 ± 0.081 μm (from 0.022 μm to 0.347 μm) all of them at 6 mm analysis diameter.

### Correlations for bilateral symmetry OS vs. OD

**Fig. 1:**
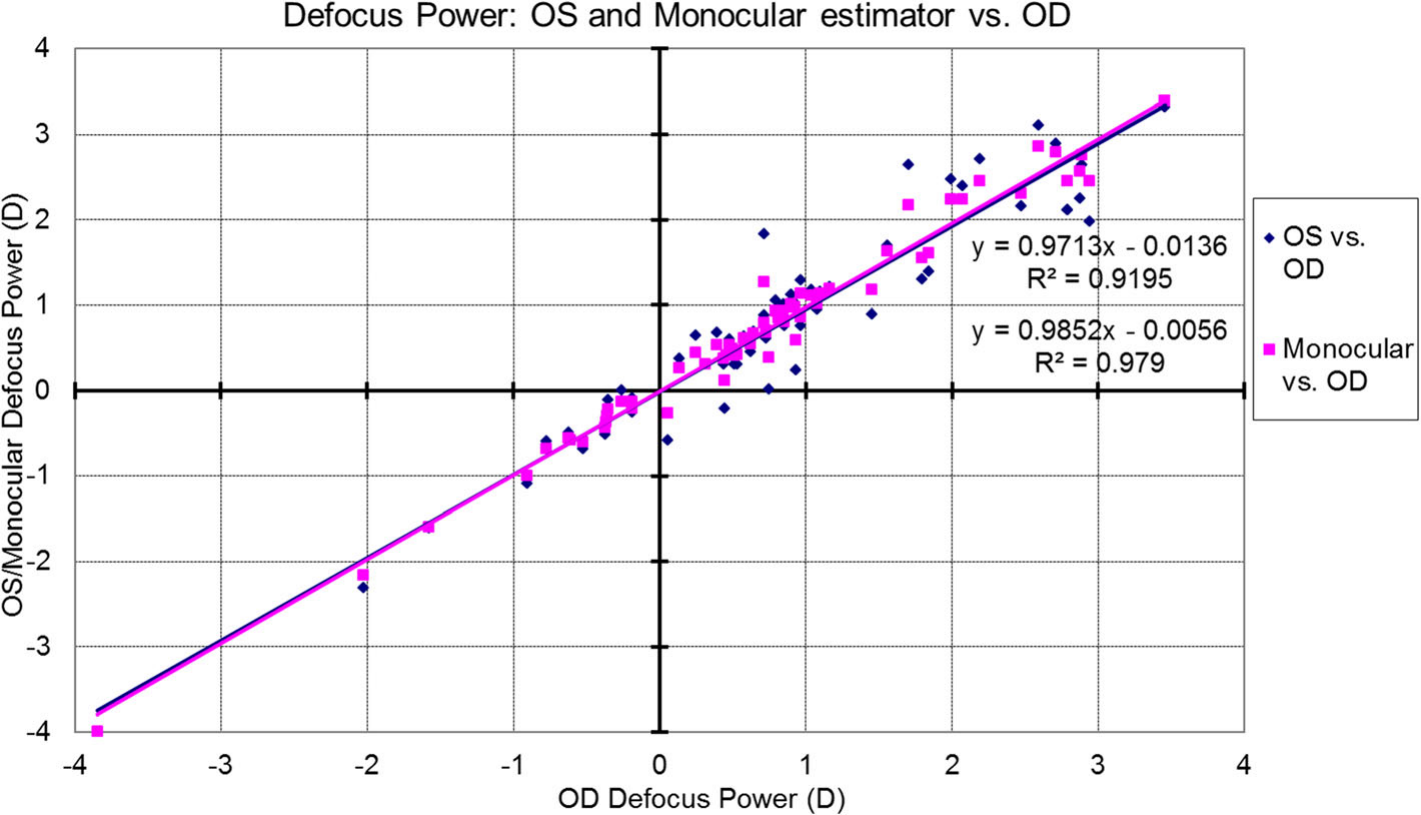
Correlation between the defocus power (spherical equivalent) in OD to OS and the monocular estimated values. Notice the excellent agreement between the three parameters, showing positive correlation (even/direct symmetry)

**Fig. 2:**
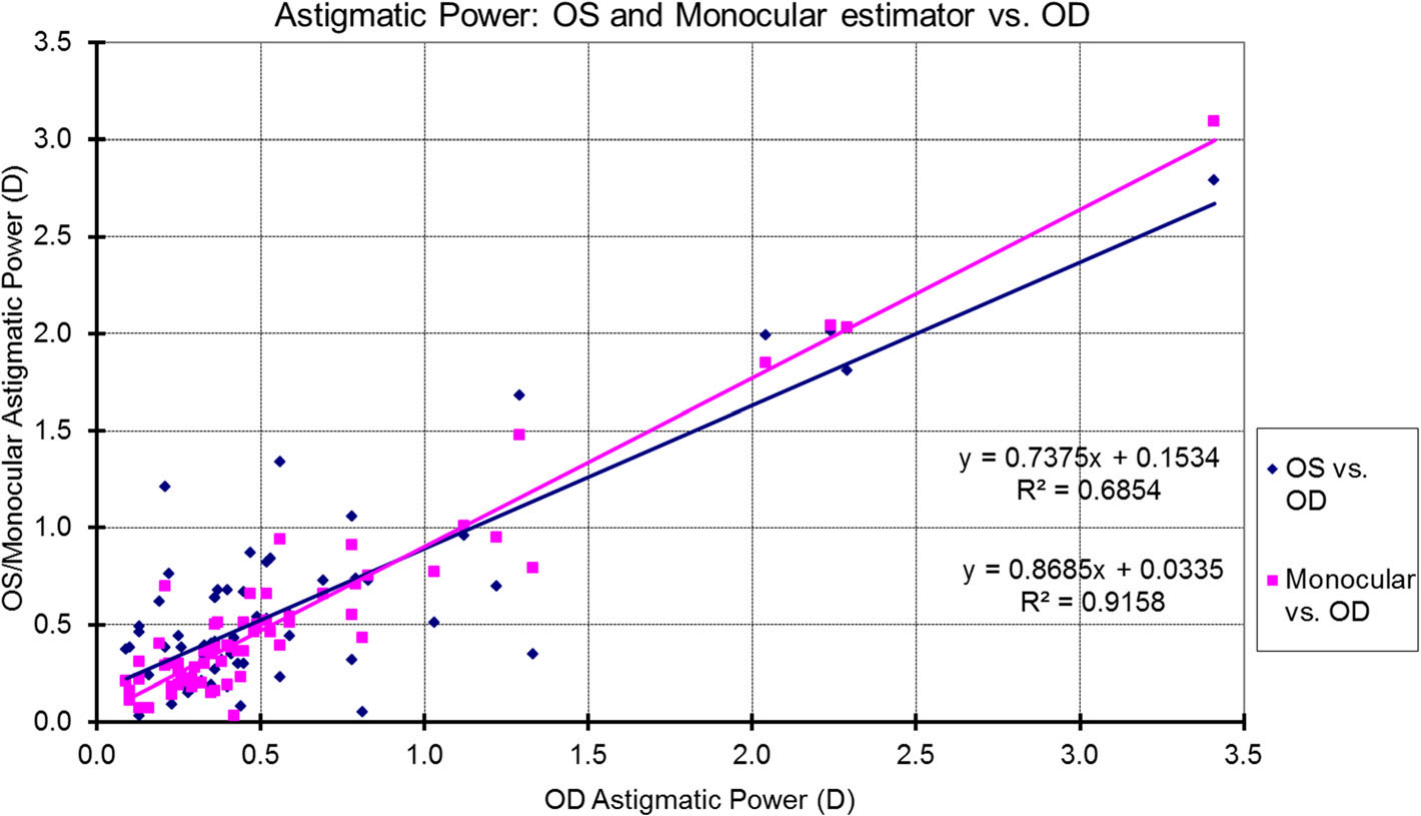
Correlation between the astigmatic power (in magnitude) in OD to OS and the monocular estimated values. Notice that the absolute astigmatism in OS was slightly smaller than in OD. However, monocular estimated values were very similar to OD, with strong positive correlation (even/direct symmetry) between OS and OD, and between OD and the monocular estimated values

**Fig. 3:**
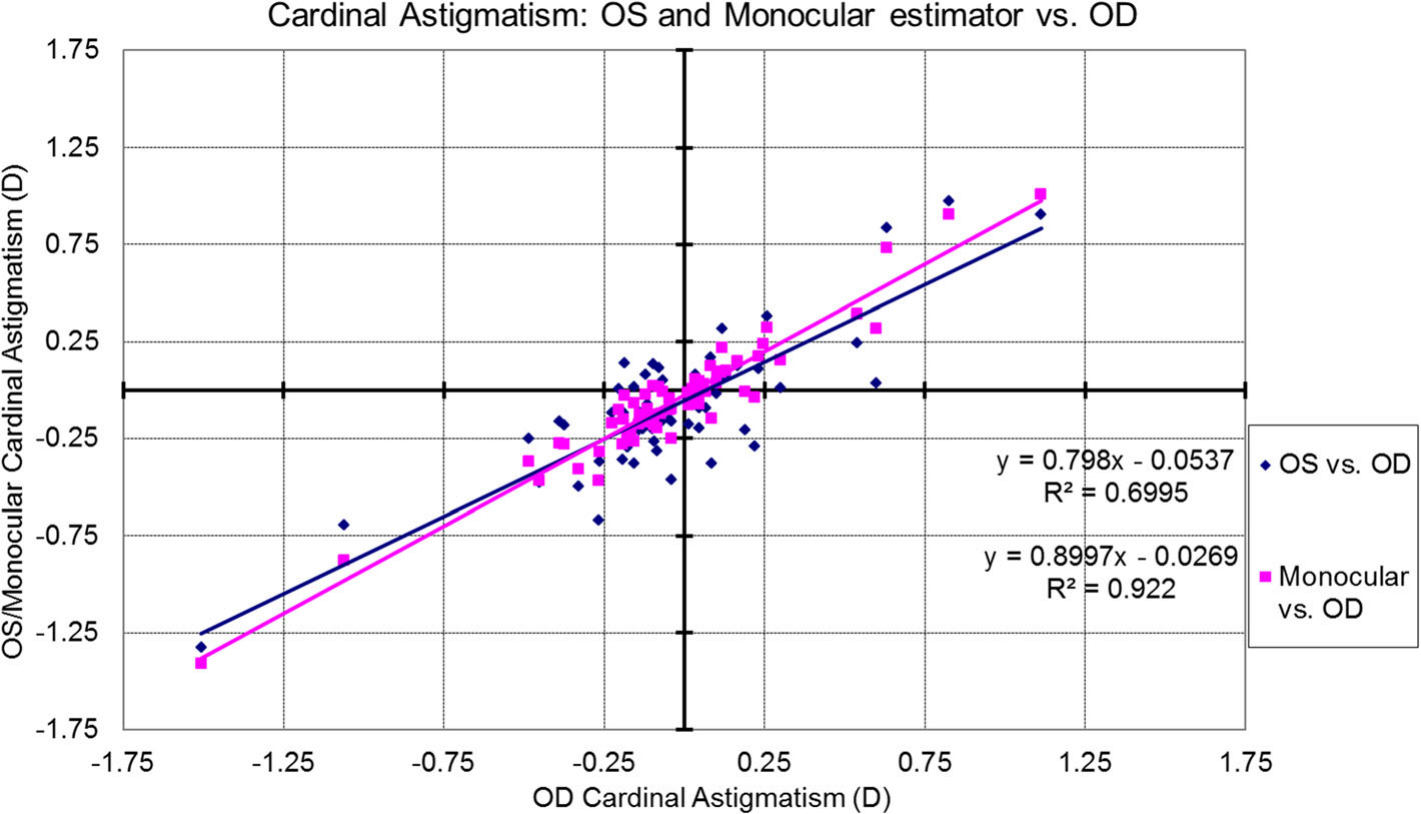
Correlation between the cardinal astigmatism in OD to OS and the monocular estimated values. The estimated monocular cardinal astigmatism, cardinal astigmatism in OD and in OS, all show very similar values and a strong positive correlation (even/direct symmetry)

**Fig. 4:**
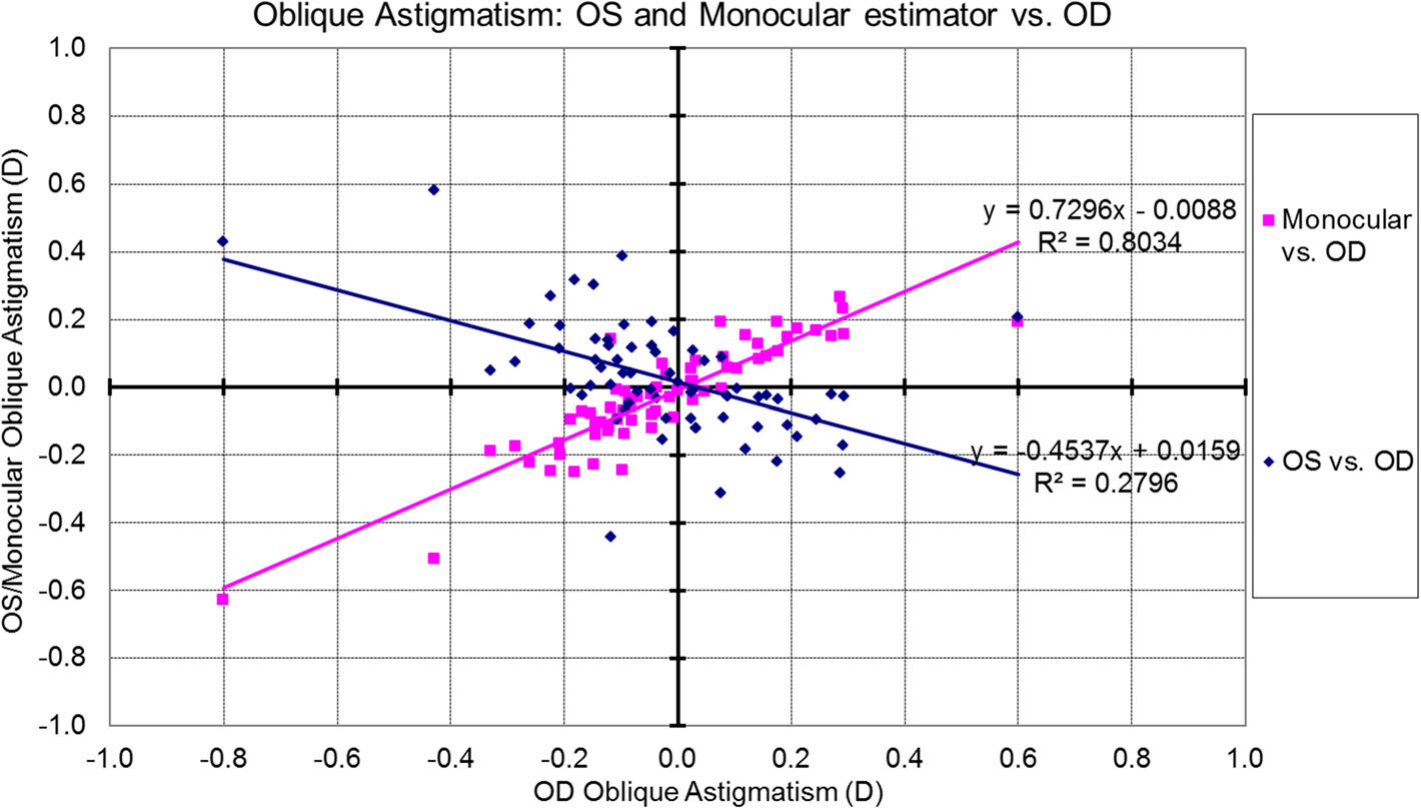
Correlation between the oblique astigmatism in OD to OS and the monocular estimated values. The monocular estimated oblique astigmatism showed strong positive correlation to OD (slope = 0.72), whilst OS showed a strong negative correlation to OD (slope = − 0.45) indicating mirror symmetry (enantiomorphism) between OS and OD for oblique astigmatism

**Fig. 5:**
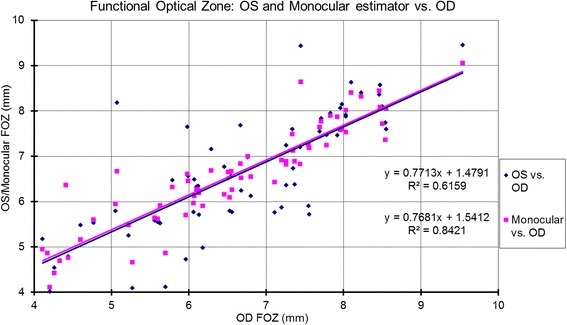
Correlation between the Functional Optical Zone (FOZ) in OD to OS and the monocular estimated values. The FOZ in OD correlated strongly and positively to both the FOZ in OS and the estimated monocular FOZ (calculated from the mirrored Zernike components of OS) showing even/direct symmetry between OS and OD in terms of FOZ

The correlations for bilateral symmetry between OS and OD based on the analyzed metrics are presented in Table 1 and Figs. 1-5. SE OD and SE OS were very similar and showed strong positive correlation (Fig. 1, R^2^ = 0.9). The absolute value of astigmatism in OS was slightly smaller than in OD (Table 1), but a strong positive correlation was seen (Fig. 2, R [2] = 0.7). Cardinal astigmatism in OD and OS was very similar and showed a strong positive correlation (Fig. 3, R^2^ = 0.7). For oblique astigmatism component, OS showed a strong negative correlation to OD (slope = − 0.45) suggesting mirror symmetry between OS and OD according to our expectation (Fig. 4). The FOZ in OD correlated strongly and positively to the FOZ in OS (Fig. 5, R^2^ = 0.62). This confirms good bilateral symmetry in corneal aberrations for our study population.

**Table 1:** The correlation between OS (oculus sinister) and OD (oculus dexter) based on spherical equivalent defocus power, astigmatism magnitude, cardinal and oblique astigmatism, and the functional optical zone

Metric	Representation	Average for OD	Range/ Median for OD	Average for OS	Range / Median for OS	Difference between OS and OD	Correlation between OS and OD
SE	Fig. 1	0.80 ± 1.30 D	−3.85 to 3.46 D / 0.73 D	0.76 ± 1.32 D	−4.15 to 3.32 D / 0.69 D	NSSD (*p* = 0.2)	SS (*p* < .0001) positive symmetry
Astigmatism (magnitude)	Fig. 2	0.57 ± 0.58 D	0.09 to 3.41 D/ 0.38 D	0.57 ± 0.52 D	0.03 to 2.79 D/ 0.41 D	NSSD (*p* = 0.5)	SS (*p* < .0001) positive symmetry
Cardinal astigmatism	Fig. 3	−0.03 ± 0.35 D	−1.51 to 1.11 D / -0.05 D	− 0.08 ± 0.34 D	−1.33 to 0.97 D/ -0.09 D	SSD (*p* = 0.02)	SS (*p* < .0001) positive symmetry
Oblique astigmatism	Fig. 4	−0.02 ± 0.20 D	− 0.80 to 0.60 D / -0.04 D	0.03 ± 0.17 D	− 0.44 to 0.58 D/ -0.00 D	NSSD (*p* = 0.09)	SS (*p* < .0001) negative symmetry
FOZ	Fig. 5	6.54 ± 1.35 mm	4.10 to 9.53 mm / 6.54 mm	6.52 ± 1.32 mm	4.01 to 9.44 mm / 6.37 mm	NSSD (*p* = 0.4)	SS (*p* < .0001) positive symmetry

*SE* = spherical equivalent; *Fig*. = figure; *FOZ* = functional optical zone; *NSSD* = no statistical significant difference; *SS* = statistical significance

### Correlations between monocular estimator and OD

The correlations between the Monocular estimator and OD based on the analyzed metrics are presented in Table 2 and Figs. 1-5. SE monocular and SE OD were very similar and showed strong positive correlation (Fig. 1, R^2^ = 0.98). The estimated monocular astigmatism (magnitude) and the estimated monocular cardinal astigmatism were very similar to OD and showed a strong positive correlation (Fig. 2, R^2^ = 0.91 and Fig. 3, R^2^ = 0.92 respectively). In terms of oblique astigmatism component and FOZ, monocular estimated values also showed strong positive correlation to OD (Fig. 4, slope = 0.73, R^2^ = 0.80 and Fig. 5, slope = 0.76, R^2^ = 0.84 respectively).

**Table 2:** The correlation between Monocular estimated values versus OD (oculus dexter) based on spherical equivalent defocus power, astigmatism magnitude, cardinal and oblique astigmatism, and the functional optical zone

Metric	Representation	Average for ME	Range / Median for ME	Difference between ME and OD	Correlation between ME and OD
SE	Fig. 1	0.78 ± 1.30 D	−4.00 to 3.39 D/ 0.68 D	NSSD (*p* = 0.2)	SS (*p* < .0001) positive symmetry
Astigmatism (magnitude)	Fig. 2	0.53 ± 0.53 D	0.03 to 3.09 D/ 0.38 D	SSD (*p* = 0.02)	SS (*p* < .0001) positive symmetry
Cardinal astigmatism	Fig. 3	−0.05 ± 0.33 D	−1.41 to 1.01 D / -0.07 D	SSD (*p* = 0.02)	SS (*p* < .0001) positive symmetry
Oblique astigmatism	Fig. 4	−0.03 ± 0.16 D	− 0.63 to 0.27 D / -0.02 D	NSSD (*p* = 0.4)	SS (*p* < .0001) positive symmetry
FOZ	Fig. 5	6.56 ± 1.13 mm	4.10 to 9.04 mm/ 6.60 mm	NSSD (*p* = 0.4)	SS (*p* < .0001) positive symmetry

*ME* = monocular estimator; *SE* = spherical equivalent; *Fig.* = figure; *FOZ* = functional optical zone; *NSSD* = no statistical significant difference; *SSD* = statistical significant difference; *SS =* statistical significance

### Correlations between the binocular estimator and monocular estimator

**Fig. 6:**
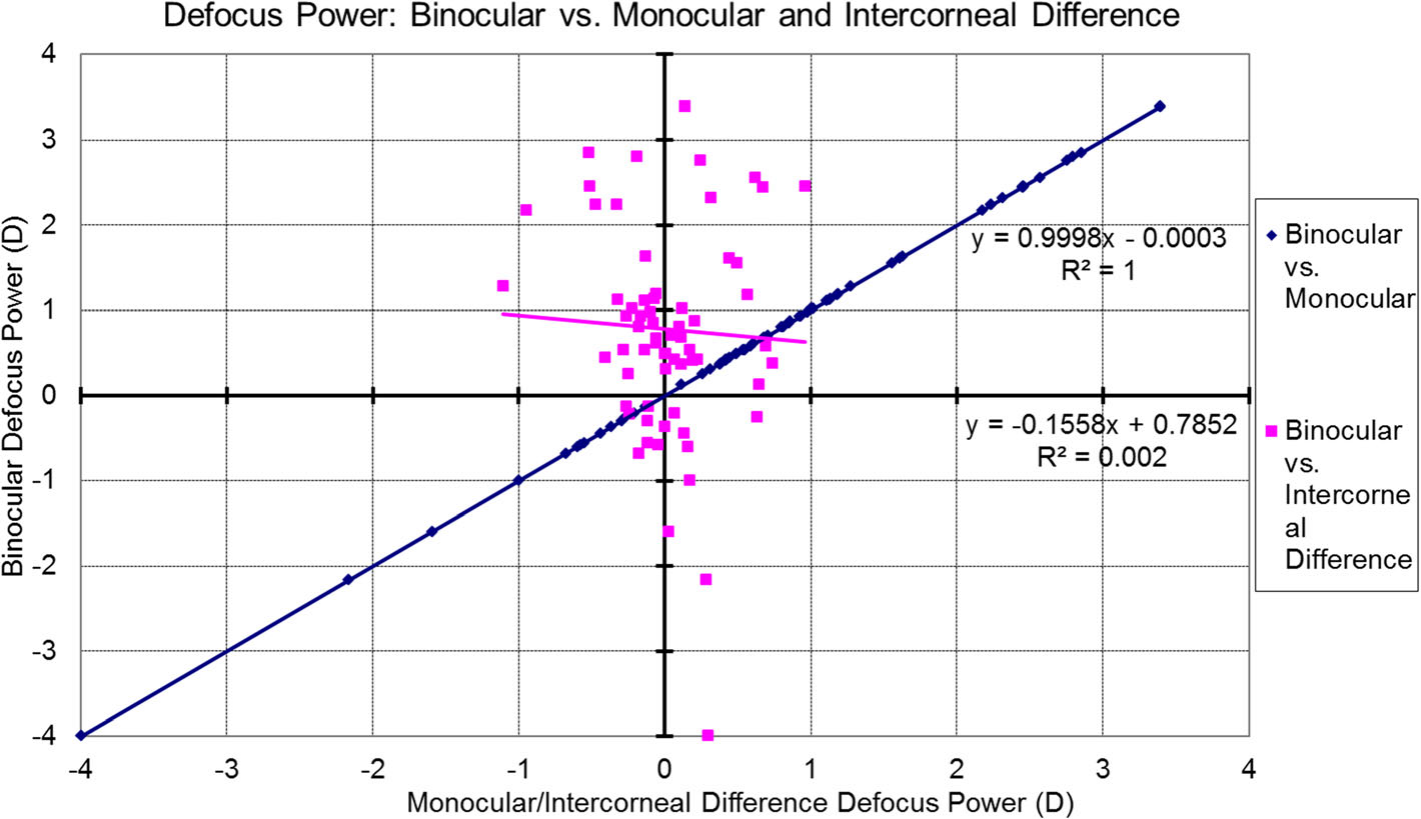
Correlation between the binocular estimated defocus power (Spherical equivalent) to the monocular estimated defocus power and the intercorneal difference in defocus power. Notice the excellent agreement between Binocular and Monocular estimated values showing positive correlation (even/direct symmetry) compared to the non-significant (*P* = 0.7) negative correlation between the Binocular estimated values and the Intercorneal differences (odd/mirrored symmetry)

**Fig. 7:**
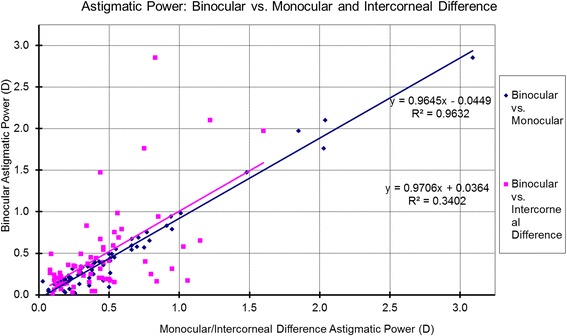
Correlation between the estimated binocular astigmatic power (magnitude) to the estimated monocular astigmatic power (magnitude) and the Intercorneal difference in terms of astigmatic power (magnitude). The absolute value of the estimated binocular astigmatism was smaller than estimated monocular astigmatism, but very similar to the intercorneal differences in astigmatism; with all three showing strong positive correlation (even/direct symmetry)

**Fig. 8:**
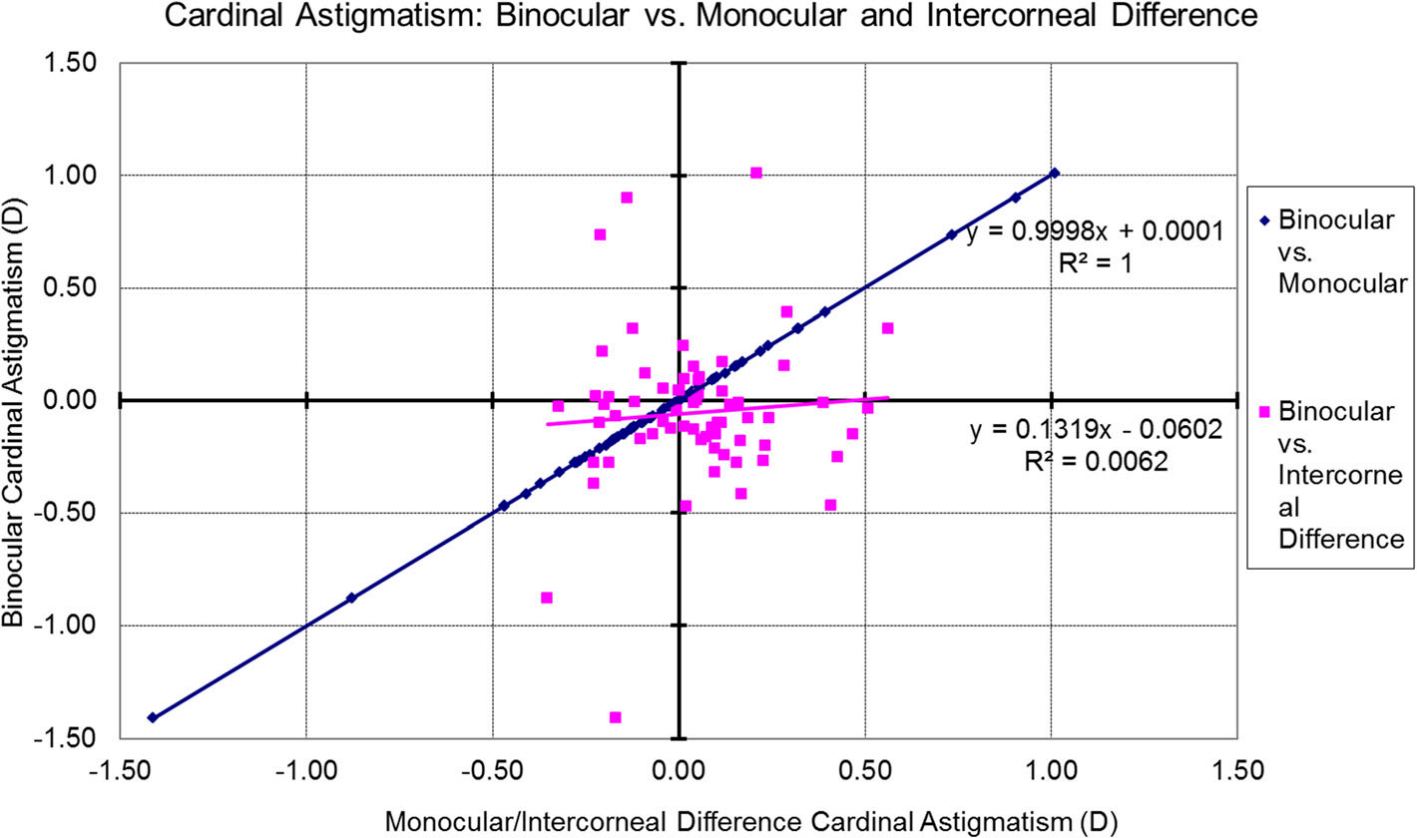
Correlation between the estimated binocular cardinal astigmatism to the estimated monocular cardinal astigmatism and the Intercorneal difference in terms of cardinal astigmatism. Binocular estimated cardinal astigmatism correlated perfectly (R2 = 1) with monocular estimated cardinal astigmatism (even/direct symmetry), however showing poor correlation with the intercorneal differences in cardinal astigmatism (R2 = 0.0062)

**Fig. 9:**
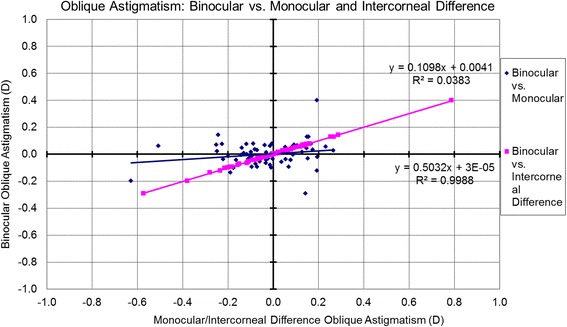
Correlation between the estimated binocular oblique astigmatism to the estimated monocular oblique astigmatism and the Intercorneal difference in terms of oblique astigmatism. The estimated binocular oblique astigmatism showed a very strong positive correlation (even/direct symmetry) to the intercorneal differences in terms of oblique astigmatism however showing a poor correlation to the estimated monocular values

**Fig. 10:**
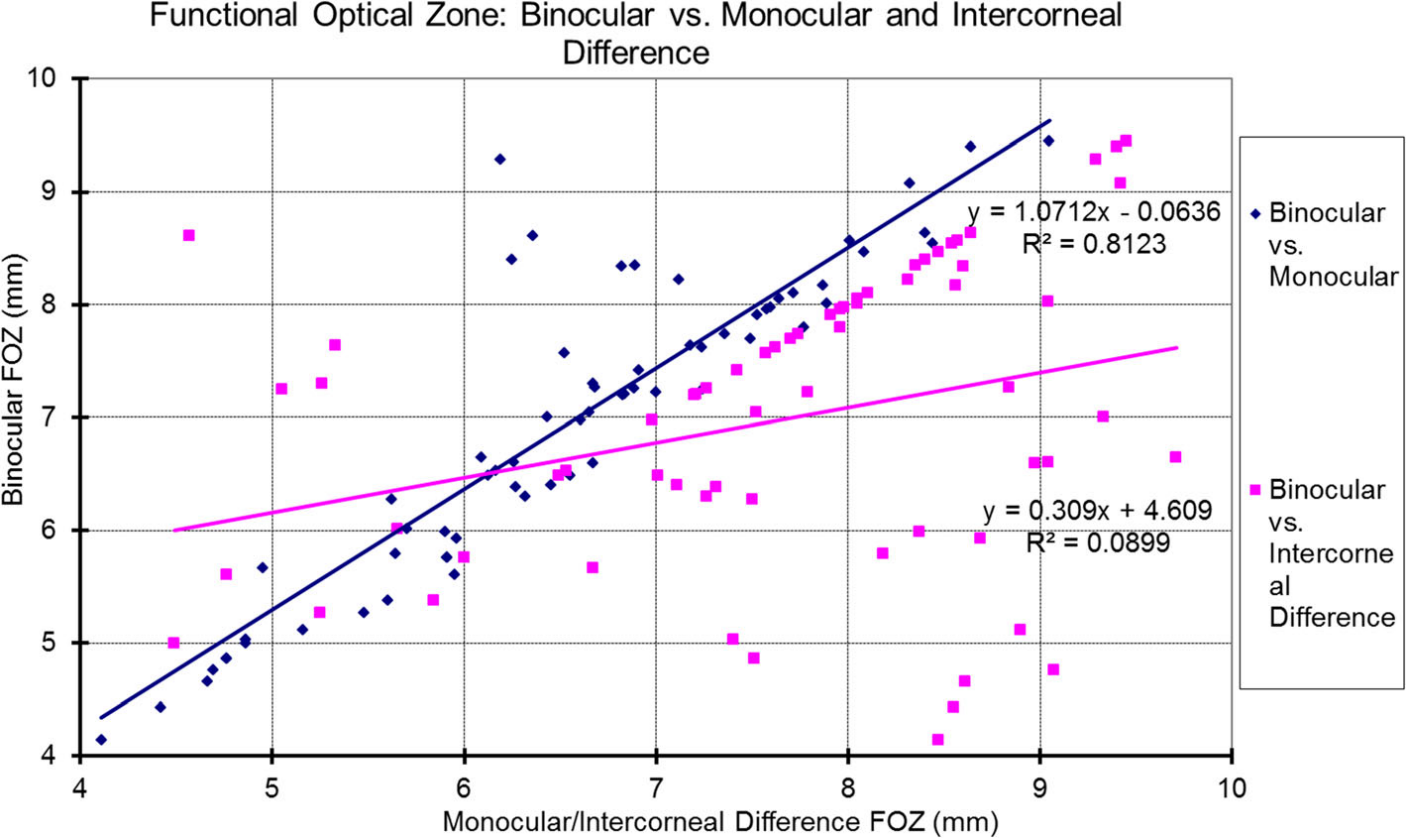
Correlation between the estimated binocular Function Optical Zone (FOZ) to the estimated monocular FOZ and the Intercorneal difference in terms of FOZ. The estimated binocular FOZ was larger than monocular FOZ, but both strongly positively correlated (even/direct symmetry). The estimated binocular FOZ was smaller than the intercorneal differences, but both strongly positively correlated (even/direct symmetry). This confirms good symmetry in corneal aberrations for our study population

The associations between the Binocular estimator and Monocular estimator based on the analyzed metrics are presented in Table 3 and Figs. 6-10. Binocular estimated SE and cardinal astigmatism correlated perfectly to monocular estimated SE and cardinal astigmatism (Fig. 6, R^2^=1 and Fig. 8, R^2^=1 respectively). The estimated binocular astigmatism (in magnitude) was smaller than estimated monocular astigmatism but showed strong positive correlation (Fig. 7, R^2^=0.96). The estimated binocular oblique astigmatism showed a weak correlation to the estimated monocular values (Fig. 9, R^2^= 0.03). The estimated binocular FOZ was larger than monocular FOZ, but both showed strong positive correlation (Fig. 10, R^2^=0.81). This could explain why usually CDVA is better binocularly than monocularly.

**Table 3:** The correlation between the Binocular and the Monocular estimated values based on spherical equivalent defocus power, astigmatism magnitude, cardinal and oblique astigmatism, and the functional optical zone

Metric	Representation	Average for BE	Range/ Median for BE	Difference between BE and ME	Correlation for BE Vs ME
SE	Fig. 6	0.78 ± 1.30 D	−4.00 to 3.39 D/ 0.68 D	NSSD (*p* = 0.2)	SS (*p* < .0001) positive symmetry
Astigmatism (magnitude)	Fig. 7	0.46 ± 0.52 D	0.02 to 2.85 D/ 0.31 D	SSD (*p* < 0.0005)	SS (*p* < .0001) positive symmetry
Cardinal astigmatism	Fig. 8	−0.05 ± 0.33 D	−1.41 to 1.01 D/ -0.07 D	NSSD (*p* = 0.3)	SS (*p* < .0001) positive symmetry
Oblique astigmatism	Fig. 9	−0.00 ± 0.09 D	− 0.29 to 0.40 D/ -0.00 D	NSSD (*p* = 0.08)	NSS (*p* = .1) positive symmetry
FOZ	Fig. 10	6.97 ± 1.34 mm	4.13 to 9.44 mm/ 7.20 m	SSD (*p* < 0. 0001)	SS (*p* < .0001) positive symmetry

*BE* = binocular estimator; *ME* = monocular estimator; *SE* = spherical equivalent; *Fig.* = figure; *FOZ* = functional optical zone; *NSSD* = no statistical significant difference; *SSD* = statistically significant difference; *SS* = statistical significance

### Correlations between binocular estimator and Intercorneal difference

The relationships between the Binocular estimator and the Intercorneal variance based on the analyzed metrics are presented in Table 4 and Figs. 6-10. Binocular estimated SE was larger and did not correlate to intercorneal differences (Fig. 6, R^2^=0.002). The estimated binocular astigmatism was similar to the intercorneal discrepancies in astigmatism and showed a positive correlation with some outliers (Fig. 7, R^2^=0.34). Binocular estimated cardinal astigmatism showed weak correlation with the intercorneal differences in cardinal astigmatism (Fig. 8, R^2^ = 0.0062). The estimated binocular oblique astigmatism showed almost perfect positive correlation to intercorneal differences (Fig. 9, R^2^=0.99). The estimated binocular FOZ was smaller than the intercorneal differences, and also showed poor correlation (Fig. 10, R^2^=0.08). This indicates bilateral symmetry in higher order aberrations in our cohort, as by definition, for intercorneal differences, horizontally symmetric higher order aberrations shall cancel each other.

**Table 4:** The correlation between the Binocular estimated values and the estimated Intercorneal differences based on spherical equivalent defocus power, astigmatism magnitude, cardinal and oblique astigmatism, and the functional optical zone

Metric	Representation	Average for ID	Range / Median for BE	Difference between ID and BE	Correlation for ID Vs BE
SE	Fig. 6	−0.04 ± 0.37 D	−1.11 to 0.96 D / 0.01D	SSD (*p* < 0.001)	NSS (*p* = 0.7) negative symmetry
Astigmatism (magnitude)	Fig. 7	0.44 ± 0.31 D	0.08 to 1.60 D/ 0.38 D	NSSD (*p* = 0.3)	SS (*p* < .0001) positive symmetry
Cardinal astigmatism	Fig. 8	0.05 ± 0.20 D	−0.35 to 0.56 D/ 0.05 D	SSD (*p* = 0.02)	NSS (*p* = .5) positive symmetry
Oblique astigmatism	Fig. 9	−0.00 ± 0.18 D	− 0.57 to 0.79 D/ 0.01 D	NSSD (*p* = 0.4)	SS (*p* < .0001) positive symmetry
FOZ	Fig. 10	7.64 ± 1.30 mm	4.48 to 9.70 mm/ 7.90 mm	SSD (*p* < 0. 005)	SS (*p* = .02) positive symmetry

*ID* = intercorneal difference; *BE* = binocular estimator; *SE* = spherical equivalent; *Fig.* = figure; *FOZ =* functional optical zone; *NSSD* = no statistical significant difference; *SSD* = statistically significant difference; *SS =* statistical significance; *NSS =* no statistical significance

## Discussion

This study aimed to evaluate bilateral symmetry between the right and left eyes regarding corneal wavefront aberrations, among normal subjects (without any ocular pathology) that have not undergone any ocular surgery. We correlated the FOZ, defocus, astigmatism power and axis, and cardinal and oblique astigmatism for binocular vs. monocular and binocular vs. intercorneal differences between the right and left eyes of the same subjects. It must be mentioned that the defocus term (also represented as SE here) was obtained from corneal topographic aberrations fit to the Zernike polynomials. This term cannot be interpreted clinically but was analyzed only to assess bilateral symmetry.

According to our expectations, SE, astigmatism magnitude, cardinal astigmatism component and FOZ showed even symmetry, while oblique astigmatism component showed odd symmetry suggesting Enantiomorphism regarding SE, astigmatism magnitude, cardinal astigmatism component and FOZ, and anti-symmetry in terms of oblique astigmatism, between the left and right eye. These results also confirm that the used monocular estimator is a good representation of the individual OD and OS values.

The use of the root mean square of the higher order aberration for assessing the FOZ makes our analysis robust and relatively insensitive to the analysis diameter of the subject. For our analyses, a threshold value of 0.375 D for determining the FOZ was arbitrarily chosen based upon the fact that with simple spherical error, for most people, degradation of resolution begins between 0.25 D and 0.50 D of defocus or astigmatism [29, 31]. The threshold of 0.375 D was taken as the mean value of this assumed range of refractive error resulting in degradation of resolution. If any other value were used, the general conclusions concerning the bilateral symmetry derived in this study would still hold. However, the numerical values could be a bit larger for threshold values larger than 0.375 D, and smaller for values below 0.375 D. We also analyzed our results with threshold values of 0.25 D and 0.50 D and found − 18% smaller FOZ and + 10% larger FOZ respectively.

We have determined the FOZ for different conditions: OD, OS, Monocular estimator, Intercorneal difference, and Binocular estimator. For each of those, the meaning of the determined FOZ is slightly different. For OD, OS, and Monocular estimator, FOZ represents the corneal diameter which is compatible to monocular CDVA + 0.05 logMAR [29] (i.e., the diameter for which the potential CDVA of the cornea can be regarded as normal). For Binocular estimator, FOZ represents the diameter which is compatible to binocular CDVA + 0.05 logMAR [29] (i.e., the diameter for which the potential CDVA of the patient can be regarded as standard). For the intercorneal difference, FOZ represents the diameter for which the difference in CDVA in OD vs. OS is compatible to half a line (i.e. the diameter for which the corneal aberrations in OD and OS can be regarded as equal due to a non-significant difference in their visual performance [29]).

It has been previously shown that wavefront RMS is a bad predictor of vision quality. Applegate et al. [32] showed that for an equal amount of RMS error, not all coefficients of the Zernike polynomial induce similar losses in high and low contrast logMAR acuity. Wavefront error concentrated near the center of the pyramid adversely affects visual acuity more than modes near the edge of the pyramid. In our methods, however, the analysis of intercorneal differences based on the FOZ was accounted from the RMS of the differential corneal wavefront aberration (RMS(ΔHOA)) instead of the differential RMS of corneal wavefront aberration (ΔRMS(HOAb)). The employed method a rigorous metric for analysis, since it accounts for any deviation (i.e., both inductions and reductions of the wavefront aberration, since both contribute positively to increase the RMS value). Furthermore, it can be mathematically demonstrated that:

6 $$ RMS\left(\Delta HOAb\right)\ge \Delta RMS(HOAb) $$

In our study, FOZ binocular was significantly larger than monocular. This finding could optimize refractive surgery outcomes, by emphasizing the use of large OZs, covering not only the scotopic pupil size and tolerance for possible decentration but also the FOZ binocular. If FOZ binocular cannot be determined, simple regression compensation could be used to increase the optical zone accordingly. In our sample population, this regression (*Planned*(*mm*) ≥ 1.0712 ⋅ *Monocular*(*mm*) − 0.064*mm*) could potentially optimize the post-operative CDVA. It is possible that the FOZ calculated in this manner could be larger than the planned OZ in refractive surgery, if it encompasses some portions of the transition zone, or even more significant than the ablation zone. Although planned OZ, transition zone, and ablation zone are parameters defined by the laser treatment algorithms, FOZ must be determined from the aberrations and may change with time because of the healing and biomechanical effects.

The major concern with the chosen methodology is that a binocular FOZ can only be determined using psychophysical tests or at least a model for binocular summation. We have taken one of the simplest models available for binocular summation, namely that binocular vision is ruled by the average of the aberrations of left and right eyes. Considering the natural enantiomorphism of left and right eyes concerning irregularities means that in our simple model of binocular summations horizontally oriented asymmetric aberration patterns tend to cancel out, while vertically oriented asymmetric aberration patterns and rotationally symmetric aberration patterns are retained.

It must be pointed that the conclusions of this study are specific to the small patient population, which cannot be considered as a representative of a large patient population, not allowing for definitive conclusions or evidence-based statements. We determined whether symmetry exist by comparing individual terms in a variety of ad hoc ways, ignoring the fact that retinal image quality for any given individual is not merely based on the sum of all terms. The analysis of bilateral symmetry and estimation of the FOZ should be ideally related to the subjects’ binocular vision status and determined psychophysically. We did not perform any specific visual tests (like stereoacuity tests) on binocular vision, despite these limitations, we were able to demonstrate OD-vs.-OS bilateral symmetry (which influences binocular summation) of the corneal higher-order wavefront aberrations’, FOZ, defocus, astigmatism power and axis, and cardinal and oblique astigmatism. Additionally, we did not analyze the effects of gender and age on the symmetry of aberrations. However, several studies indicate that mirror symmetry between eyes is unaffected by age or gender [33]. It must also be noted that the presented findings cannot be extrapolated to subjects with symptoms of amblyopia [34], anisometropia [35], nystagmus [36], or aniseikonia [26] without further studies; furthermore, the methodology must be adapted to estimate the FOZ for these patients.

It can be argued that the measurement technique used in this study imposes restrictions on the FOZ size, which may have underestimated value for decentred pupils. On the other hand, the data may not fit well to the Zernike polynomials up to the 7th radial order (36 Zernike coefficients). It is known that the residual irregularity of the cornea not fit by Zernike polynomials may have a significant impact on the visual quality. Ignoring this effect might bias the size of the determined FOZ, leading to a potential overestimation that can be significant.

With the currently available means, we cannot precisely evaluate the role of aberrations monocularly (subjects with a high level of irregularities can have excellent visual acuity and vice versa [37]), it is even more challenging to do it binocularly [38, 39]. The vital question in binocular vision is “the role of interocular-differences” (presented in our study as intercorneal differences), and if they can influence significantly the binocular performance. In our study, intercorneal differences were minor with the largest FOZ, lower SE values, and lower cardinal astigmatism component values. Further studies shall help to determine the impact of intercorneal differences on binocular visual performance.

An approach like ours was used by Tabernero et al. [29], applied in a different way. They analyzed directly on the cornea the FOZ in subjects pre and postoperatively. Cuesta et al. [28] found that even differences in corneal asphericity might affect the binocular visual function by diminishing the binocular contrast-sensitivity function.

Jiménez et al. [9] found that binocular function deteriorates more than monocular function after laser-assisted in situ keratomileusis (LASIK), and this deterioration increases as the interocular differences increase regarding aberrations and corneal shape. They found that interocular differences above 0.4 μm of the RMS for a 5-mm analysis diameter, lead to a decrease of more than 20% in binocular summation. Partal and Manche [40] observed a reduction from FOZ of 6.50-mm to 6.00-mm after LASIK, over a large sample of eyes in moderate compound myopic astigmatism, using direct topographic readings. Qazi et al. [41] observed a reduction from FOZ of 6.50-mm to 5.61-mm after LASIK, in a sample of eyes similar to ours, although using a different approach.

Mok and Lee [42] reported that larger optical zones decrease postoperative high-order aberrations. They found the measured high-order aberrations to be less in eyes with larger optical zones. Assessing the quality of vision (rather than the quality of the optical zone) after a refractive procedure is a separate issue. The relationship between pupil size and vision after refractive surgery is critical, and this link cannot be evaluated accurately with a measurement of aberrations through a predetermined aperture with an aberrometer. Pupil sizes vary considerably among subjects depending on light level and age [43]. Mok and Lee [42] have shown a strategy for planning optical zone size based on patient pupil size. However, an aberration analysis that considers the variations in the planned optical zone size may provide more insight into the quality of the obtained outcome.

## Conclusions

In conclusion, our results suggest that wavefront aberration can be a useful metric for the analysis of the FOZ of the cornea or the entire eye by setting appropriate limit values. This study demonstrated that FOZ monocular was smaller than FOZ binocular, while both were smaller than FOZ intercorneal (the diameter of glare-free vision [29] is larger in binocular than monocular conditions). Furthermore, reasonable bilateral symmetry was demonstrated between the eyes (which influence binocular summation [28]) related to the corneal wavefront aberrations. These findings could help optimize refractive surgery outcomes, by emphasizing the use of large OZs, also covering the FOZ binocular. Additionally, the presented simple regression compensation could be used to increase the optical zone if the binocular FOZ cannot be evaluated, however with caution, considering the similarities to our study population.
